# Influences of cognitive control on number processing: New evidence from switching between two numerical tasks

**DOI:** 10.1177/17470218231154155

**Published:** 2023-03-07

**Authors:** Andreas Schliephake, Julia Bahnmueller, Klaus Willmes, Iring Koch, Korbinian Moeller

**Affiliations:** 1 Leibniz-Institut für Wissensmedien, Tübingen, Germany; 2 Centre for Mathematical Cognition, Loughborough University, Loughborough, UK; 3 Department of Neurology, University Hospital, RWTH Aachen University, Aachen, Germany; 4 Institute of Psychology, RWTH Aachen University, Aachen, Germany; 5 LEAD Graduate School & Research Network, University of Tübingen, Tübingen, Germany

**Keywords:** Number processing, cognitive control, task switching, distance effect, SNARC effect

## Abstract

A growing body of research suggests that basic numerical abilities such as number magnitude and number parity processing are influenced by cognitive control. So far, however, evidence for number processing being influenced by cognitive control came primarily from observed adaptations to stimulus set characteristics (e.g., ratio or order of specific stimulus types) and switches between a numerical and non-numerical task. Complementing this previous research, the present study employed a task switching paradigm exclusively involving numerical tasks (i.e., magnitude comparisons and parity judgements) to examine how cognitive control processes influence number processing. Participants were presented with a single-digit number and had to either judge its parity or compare its magnitude with a standard of 5, depending on a preceding cue. Based on previous results, we expected the numerical distance effect and the spatial–numerical association of response codes (SNARC) effect to be modulated in switch trials requiring the exertion of cognitive control. Partly in line with our expectations, the numerical distance effect was reduced in switch trials. However, no modulation of the SNARC effect was observed. The results pattern suggests that number processing is influenced by cognitive control, depending on task requirements and the type of numerical information (i.e., numerical magnitude vs spatial association of numbers) that is processed. To reconcile the present and previous results, we propose an information prioritisation account, suggesting that cognitive control primarily influences the processing of the information type that requires the most explicit processing.

Over the past decade, accumulating evidence suggests that number magnitude processing is under cognitive control (e.g., Schliephake et al., 2020). Cognitive control denotes the ability of the individual “to configure itself for the performance of specific tasks through appropriate adjustments in perceptual selection, response biasing, and the online maintenance of contextual information” (Botvinick et al., 2001, p. 624). Cognitive control as one essential ability facilitating the execution and development of numerical skills has seen increasing research interest recently (e.g., Cowan & Powell, 2014; Hohol et al., 2017; Leibovich et al., 2017; Passolunghi & Lanfranchi, 2012; Peng et al., 2016; Schliephake et al., 2020). In particular, better understanding of the association between numerical and cognitive control processes might help broaden our understanding of how number processing is embedded into more general cognitive systems (e.g., Klein et al., 2016). Of particular interest for the present study are previous findings indicating that cognitive control influences basic numerical processes as simple as number magnitude processing (e.g., Huber et al., 2013; Huber, et al., 2014; Macizo & Herrera, 2011, 2013; Moeller et al., 2013; Pfister et al., 2013).

Notably, only a few studies looked at whether and how basic numerical processing is affected in settings requiring the exertion of cognitive control, such as when switching between tasks (Schliephake et al., 2021; Wendt et al., 2013). Therefore, the present study employed a task switching paradigm to evaluate both the generalisability and specificity of cognitive control influences on numerical processing across different task combinations. In the following, we briefly review existing evidence regarding the influence of cognitive control on number processing before describing the present study in more detail.

## Cognitive control effects of stimulus set characteristics in number processing

So far, most evidence regarding cognitive control influences on basic number processing was derived from findings that indicate that participants adapt to stimulus set characteristics, such as the relative frequency of incongruent to congruent stimuli in different experimental conditions (e.g., 25%, 50%, and 75% incongruent items, Macizo & Herrera, 2013; see also Moeller et al., 2013). Adaptations to stimulus set characteristics were observed for different numerical effects such as the unit-decade compatibility effect, the numerical distance effect, and the SNARC effect (e.g., Huber et al., 2013, 2014, 2016; Macizo, 2017; Macizo & Herrera, 2011, 2013; Moeller et al., 2011; Pfister et al., 2013).

Of particular interest for the present study is first evidence of an influence of cognitive control on basic number magnitude processing as indexed by the numerical distance effect. The numerical distance effect (Moyer & Landauer, 1967) reflects the finding that comparisons of numerically closer numbers (e.g., 4 and 5) are slower and more error-prone than comparing more distant numbers (e.g., 5 and 9). First evidence for an influence of cognitive control on the distance effect comes from a study by Moeller and colleagues (2013), who found that the distance effect in two-digit number comparison was modulated by combinations of specific digits at the unit-digit position. For instance, when distance 2 was specifically associated with unit digits 9 and 1 in number pairs crossing a decade boundary (e.g., 49_51), responses were faster compared with other number pairs with a larger distance also crossing a decade boundary (e.g., 45_53 with a distance of 8). The authors interpreted this finding as evidence for adaptations to stimulus characteristics through cognitive control processes (see also Pinhas et al., 2015; Pinhas et al., 2010, for an influence of stimulus characteristics related to the distribution of digits in the set). In the current study, we investigated whether the numerical distance effect is modulated through task switching—a task typically reflecting cognitive control demands—to provide converging evidence for influences of cognitive control on number magnitude processing.

Similar to the numerical distance effect, it was also shown that the spatial–numerical association of response codes (SNARC) effect is influenced by stimulus set characteristics (i.e., the order in which stimuli are presented). The SNARC effect reflects faster responses to comparably smaller numbers with the left as compared with the right hand and faster responses to comparably larger numbers with the right as compared with the left hand. Dehaene and colleagues (1993, see also Wood et al., 2008 for a meta-analysis) argued that the SNARC effect indicates a spatial dimension in the representation of number magnitude (but see van Dijck & Fias, 2011). Furthermore, there is also first evidence indicating that inter-individual variation in the SNARC effect may at least partially be explained by cognitive control-related domain-general abilities, such as inhibition (e.g., Hoffmann et al., 2014; Schroeder et al., 2016; see Schliephake et al., 2022 for evidence from a task switching paradigm).

Concerning direct influences of cognitive control on the SNARC effect, Pfister and colleagues (2013) found that the SNARC effect was modulated by the SNARC-congruency of the response required in the preceding trial. Such modulations, referred to as congruence sequence effects, are thought to be caused by a conflict–control loop (e.g., Botvinick et al., 2001; Egner, 2007) which elicits adaptations of information processing through cognitive control (for reviews, see Egner, 2007; Schuch et al., 2019). In particular, after an incongruent trial (i.e., requiring participants to respond with the left hand when the presented number was larger than 5 or with the right hand when it was smaller than 5), the SNARC effect was reduced compared with trials in which the preceding trial was congruent (i.e., requiring participants to respond with the right hand when the number was larger or with the left hand when the number was smaller than 5). This difference in the size of the SNARC effect due to SNARC congruency of the preceding response indicates that the switching between congruent and incongruent items influences spatial–numerical associations and that participants seemingly adapt to the congruency of items on a trial-by-trial basis. Pfister and colleagues (2013) suggested that the sequential modulation of the SNARC effect indicates the flexible and adaptive accessibility of spatial–numerical associations and thus indicates an influence of cognitive control on the SNARC effect. The current study aimed to investigate whether the distance effect and the SNARC effect are affected by a more active exertion of cognitive control as required in task switching.

## Cognitive control effects of task switching on number processing

In task switching, at least two tasks have to be performed. Tasks are either being repeated or switched in consecutive trials (i.e., repetition vs switch trials). When switching between tasks, a common finding is that the processing speed of either task is reduced, and more errors are committed (Jersild, 1927; Spector & Biederman, 1976). This is referred to as switch costs. Such switch costs were found for various task switching paradigms and, hence, are considered a robust phenomenon (e.g., Kiesel et al., 2010; Koch et al., 2018; Vandierendonck et al., 2010). Switch costs allow the assessment of cognitive control processes that may affect the processing of basic numerical information (Koch et al., 2010).

Previous studies employing numerical stimuli in task switching paradigms were mostly interested in investigating cognitive control as reflected by the mechanisms underlying task switching (e.g., Arrington & Logan, 2005; Kiesel et al., 2007; Koch et al., 2004; Logan & Bundesen, 2004; Schneider & Logan, 2005). For instance, potential causes of switch costs (e.g., processes of bottom**-**up vs top**-**down control, e.g., Arrington & Logan, 2005) and effects of different cue types were evaluated in the task switching paradigm using numerical stimuli (e.g., Kiesel et al., 2007; Schneider & Logan, 2005).

However, only few studies addressed interactions of number processing and cognitive control in task switching situations. For instance, Declerck et al. (2012) used a language switch paradigm employing cued digit naming in either the first language or in the second language. The authors found that language switch costs are larger for smaller numerical distances as compared with large numerical distances (see also Contreras-Saavedra et al., 2020, for similar evidence). This implies an interaction of number magnitude processing and language selection processes in bilinguals (Declerck et al., 2012). Moreover, Schliephake et al. (2020) reported evidence for a modulation of the numerical distance effect employing a magnitude comparison task in an input-output modality compatibility switch paradigm (see Stephan & Koch, 2010, 2011 for a detailed description of the paradigm). In the employed paradigm, participants had to switch between either compatible modality pairs (e.g., visual input–manual output or auditory input–vocal output) or incompatible modality pairs (visual input–vocal output or auditory input–manual output). The authors found that the numerical distance effect was increased when input–output modality compatibility switched. To account for this finding, Schliephake and colleagues (2021) proposed that concurrent processing demands of semantic number magnitude information and input–output modality may lead to reduced processing efficiency of number magnitude information, which was reflected by a larger distance effect.

To the best of our knowledge, only one study investigated whether the SNARC effect was influenced by task switching. Wendt and colleagues (2013) found a larger SNARC effect in switch trials when participants switched between classifying a digit as odd or even and judging whether a letter was a vowel or consonant. Thus, when switching from a numerical task to a letter processing task, an effect of cognitive control on spatial numerical associations was observed as reflected by the modulation of the SNARC effect. Wendt and colleagues (2013) argued that, in switch trials, the repetition of a previous response might be actively suppressed to reduce the risk of wrong re-execution, which in turn may have caused the observed increase of the SNARC effect.

## The present study

The present study aimed to evaluate whether specific task combinations affect how cognitive control interacts with basic number processing. In particular, we aimed to conceptually replicate and generalise previous findings regarding task switching on the numerical distance effect and the SNARC effect (Schliephake et al., 2020; Wendt et al., 2013). In contrast to previous paradigms that mixed numerical tasks with input–output modality compatibility (Schliephake et al., 2020) or with letter classification (Wendt et al., 2013), in the current study, participants had to switch between two numerical tasks (magnitude comparisons and parity judgements) using identical stimuli for both tasks.

In line with findings reported by Wendt and colleagues (2013) indicating a larger SNARC effect in switch trials than in repeat trials, we expected to observe the SNARC effect to be increased when active exertion of cognitive control is required. In particular, domain-general cognitive resources (e.g., monitoring, working memory updating, inhibition, etc., cf. Miyake et al., 2000), which are required to resolve conflicts in incongruent SNARC trials, should be reduced in switch trials due to their involvement in task switching. In turn, we expected an increased SNARC effect (i.e., the difference between SNARC-congruent and incongruent trials) in switch trials compared with repetition trials.

The predictions are less clear for the numerical distance effect. On one hand, and similar to the findings by Schliephake et al. (2020), one may expect the numerical distance effect to be increased in task switches compared with repetitions. This would reflect a similar influence of cognitive control on the processing of number magnitude information as observed for its spatial association (reflected by the SNARC effect). On the contrary, behavioural results regarding the effects of cognitive control on number magnitude processing by Moeller et al. (2013) and evidence from computational modelling by Huber et al. (2013, 2016), on influences of passive adaptations to stimulus set characteristics rather suggest numerical distance effects to be reduced when cognitive control is required. The latter would indicate differential influences of cognitive control on different numerical effects.

## Method

### Participants

The data of 59 out of 64 participants tested were considered for analysis (45 women, 14 men, *M_age_* = 24.7 years, *SD* = 4.3 years). Data from 5 participants were excluded because they either committed more than 30% errors overall (50% guessing rate; 3 participants) or because they committed more than 50% errors in at least one block (2 participants). All participants were university students from different majors and reported normal or corrected to normal visual acuity. Participation was voluntary and was compensated with 6 € or course credit. The local ethics committee of the Leibniz-Institut für Wissensmedien, Tübingen, Germany approved the study.

### Task, stimuli, and procedure

Participants had to categorise single-digit numbers as either smaller or larger than 5 (magnitude comparison task) or as odd or even (parity judgement task). Participants were asked to complete three blocks of trials: one single-task block requiring only magnitude comparisons, one single-task block requiring only parity judgements, and a third task switching block involving both magnitude comparison and parity judgement trials. The order of these three blocks was counterbalanced across participants.

Responses in all three blocks had to be given manually by pressing either the “L” or “A” key of a standard QWERTZ keyboard using the right and left index finger, respectively. Instructions emphasised both response accuracy and speed. Each block was split into two across participants orthogonally counterbalanced halves for which the hand-to-response assignment was reversed. This ensured that “larger” and “smaller” as well as “even” and “odd” responses were balanced across response hands. Thus, for half of a block, participants had to press the “A” key for “smaller”/“odd” decisions and the “L” key for “larger”/“even” decisions. For the other half of a block, participants had to press the “L” key for “smaller”/“odd” decisions and the “A” key for “larger”/“even” decisions. The order of hand-to-response assignments was counterbalanced across participants.

In all three blocks, Arabic digits 1–4 and 6–9 served as stimuli. Each single-task block (parity judgement or magnitude comparison) had 112 experimental trials, displaying each number 14 times; this means 7 times per hand to response assignment. In the switch block, each stimulus was displayed 28 times per half, 14 times in a parity judgement, and 14 times in a magnitude comparison task. Thus, the task switching block had 448 experimental trials, displaying each number 56 times altogether. Each task switching block consisted of 224 repetition and switch trials, respectively. Stimuli were presented in Times New Roman font, size 20 (resulting in a height of approximately 2.5 cm) in black against a white screen. Stimuli appeared at the centre of a 21-in. computer monitor driven at a resolution of 1920 × 1200 pixels. The viewing distance was approximately 60 cm.

Stimuli were pseudo-randomised so that the same number never occurred twice in a row. Moreover, numbers with the same numerical distance to 5 (e.g., 4 and 6 both have a distance of 1) never occurred more than two times in a row. In switch blocks, there was an identical number of parity judgements, magnitude comparisons, task switches, and task repetitions. Crucially, all transition types (i.e., switch–switch, switch–repetition, repetition–switch, and repetition–repetition) were pseudo-randomised so that repetition trials and switch trials did not occur more than four times in a row. The number of repetition- (e.g., a sequence of items all requiring magnitude comparison) and switch trials (e.g., a sequence of items switching between magnitude comparison and parity judgement from item to item) was limited to 4. This was chosen deliberately based on previous studies investigating cognitive control in number processing (i.e., Schliephake et al., 2022). Overall, 32 pseudo-random trial sequences were generated prior to the experimental session. Each pseudo-random trial sequence was employed twice.

**Figure 1.**
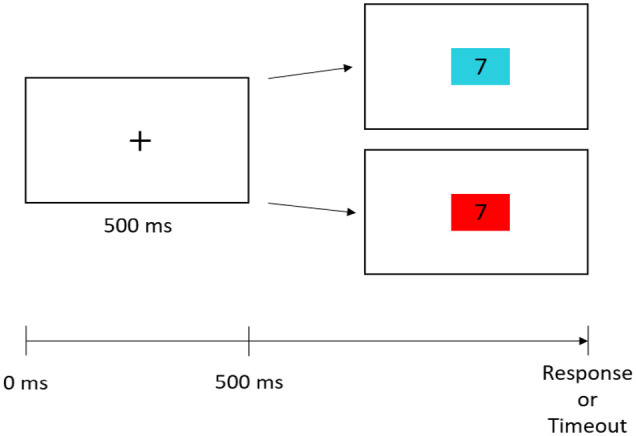
Exemplary depiction of a switch block trial. The fixation cross was displayed for 500 ms. Then the participant either had to judge the parity (cued by a turquoise rectangle) or compare the magnitude of a presented number with the standard 5 (cued by a red rectangle). A trial ended after a response button was pressed or was timed out after 4,000 ms.

In both single-task- and switch blocks, each experimental trial started with a fixation cross-displayed for 500 ms, followed by stimulus presentation. In addition, in trials of the task switching block, a cue was presented simultaneously with and for the same duration as the numerical stimulus, indicating whether a parity judgement or magnitude comparison had to be performed on the respective number. The cue was either a red or a cyan square (6 cm × 4 cm) with the stimulus located at its centre. A red square indicated that a magnitude comparison had to be performed on the number presented, while a cyan square indicated a parity judgement in the current task. A trial was ended when a response was given or was timed out automatically after a maximum response interval of 4,000 ms. Time intervals from stimulus onset until one of the response keys was pressed were considered as RTs. A response was followed by an inter-stimulus interval of 500 ms Figure 1.

After each change in the hand-to-response assignment (i.e., at the beginning and in the middle of each single-task and switch block), participants had to perform three supervised practice trials. Each participant completed the experiment in one session that lasted approximately 50 min.The experiment was programmed using Experiment Builder software (SR Research, Ottawa, Canada).

### Design and analyses

To investigate potential modulations of the distance effect and the SNARC effect resulting from *task switching*, a series of three 2 × 2 within-subject ANOVAs was conducted. Significant ANOVA interactions were followed-up by univariate ANOVAs to assess simple effects. In addition, a Bayesian analysis approach (Masson, 2011) was applied to allow for inferences about evidential support for the null hypothesis being true (i.e., that there is no difference in the SNARC- or distance effect between conditions). For the interpretation of Bayes factors, we considered the classification suggested by van Doorn and colleagues (2019). This classification differentiates between strong (BF_01_ < 1/10), moderate (1/10 < BF_01_ < 1/3), and inconclusive evidence against H_0_ (1/3 < BF_01_ < 3) as well as moderate (3 < BF_10_ < 10) and strong evidence in favour of H_1_ (BF_10_ > 10). Analyses presented here focused on RT as the dependent variable. To approximate normal distribution, RT data were log-transformed prior to the analysis, but raw reaction times were reported when describing significant results. The data and analysis scripts are available from the authors upon request.

A sensitivity analysis was conducted using More Power (Campbell & Thompson, 2012) to determine the minimal effect size for the interaction term in a 2 × 2 within-subject repeated-measures ANOVA. The sensitivity analysis indicated that with a sample size of N = 60 and alpha = .05 a large effect of η_p_^2^ = .12 or larger is detectable with a statistical power of 0.8.

Regarding the distance effect in number comparison trials, an ANOVA was conducted to evaluate the effects of the factors *numerical distance* (small vs large) and *task switching* (repetition vs switch trials). Similarly, concerning the SNARC effect in magnitude comparison trials, an ANOVA was conducted, which included the factors *SNARC congruency* (congruent vs incongruent) and *task switching* (repetition vs switch trials). A trial is considered SNARC congruent in case a left-hand response is required for a small number and a right-hand response for a large number. Vice versa, a trial is considered SNARC incongruent in case a left-hand response is required for a large number and a right-hand response for a small number. The same ANOVA was run again on data from parity judgement trials to evaluate the modulation of the SNARC effect when numerical magnitude processing was not required for the task at hand.

## Results

Data preparation and analyses were done using the statistical software R 3.6.1 (R Core Team, 2020). Prior to the RT analyses, practice trials, error trials,^1^ and non-responses (due to time out) were eliminated. Fixed cut-offs of RT < 200 ms and RT > 2,000 were applied to eliminate accidental keypresses or extraordinary long responses, resulting in a loss of 4.4 % of the data. Moreover, RT outliers deviating more than ±3 *SD* from an individual’s mean reaction time were also removed from the data set. These data pre-processing steps resulted in a total loss of about 9.0 % of the data. No evidence for a modulation of numerical effects due to *task switching* was observed in the ER analyses. To still provide the interested reader with a brief overview of RTs and ERs of all conditions, we report the means and standard deviations of the respective numerical effects in the online Supplementary Material. The following results evaluate modulations of distance- and SNARC effects by task switching using RT as dependent variable.

### Modulation of the distance effect in magnitude comparison

Considering trials requiring magnitude comparisons only, an ANOVA with the within-subject factors *numerical distance* (small vs large) and *task switching* (repetition vs switch trials) was run on RT. A significant main effect of numerical distance on RT was observed, *F*(1, 58) = 85.34, *p* < .001; η_p_² = .60, with mean RT being shorter for large numerical distances (*M* = 815 ms, *SD* = 154 ms) than for small numerical distances (*M* = 862 ms, *SD* = 151 ms). Furthermore, distance effects were positive in 49/59 participants (83.1%). In addition, *task switching* (repetition vs switch) had a significant main effect, *F*(1, 58) = 166.97, *p* *<* .001; η_p_² = .74, with significantly longer reaction times in switch trials (*M* = 910 ms, *SD* = 176 ms) as compared with the repetition trials (*M* = 773 ms, *SD* = 140 ms). In total, 57/59 (96.6%) of the participants showed longer reaction times in switch trials.

The interaction of *numerical distance* and *task switching* was significant [*F*(1, 58) = 4.03, *p* = .049; η_p_² = .06]. The distance effect was larger in repetition trials (*M* = 54 ms, *SD* = 65 ms) as compared with switch trials (*M* = 38 ms, *SD* = 48 ms). Simple effects indicated that the distance effect was significant in both repetition, *F*(1, 58) = 41.22 *p* < .001; η_p_² = .41 see above, and switch trials, *F*(1,58) = 37.61, *p* < .001; η_p_² = .39. Distance effects were positive in 50/59 participants (86.7%) in repetition trials and in 46/59 participants (78.0%) in switch trials. Bayesian analyses revealed that the Bayes factor for the null hypothesis was BF_01_ = 0.130, reflecting moderate evidence against a null effect of task switching on the numerical distance effect.

### Modulation of the SNARC effect in parity judgement

Evaluating trials requiring parity judgements only, the ANOVA with the within-subject factors *SNARC congruency* (congruent vs incongruent) and *task switching* (repetition vs switch trials) was run on RT. The main effect of *SNARC congruency* was significant, *F*(1, 58) = 7.18, *p* = .01; η_p_² = .11, indicating a regular SNARC effect with mean RTs being shorter for congruent trials (*M* = 851 ms, *SD* = 154 ms) as compared with incongruent trials (*M* = 870 ms, *SD* = 175 ms). The SNARC effect was positive in 32/57 participants (56.1%). *Task switching* (repetition vs switch) also had a significant main effect on RT, *F*(1,58) = 359.07, *p* *<* .001; η_p_² = .86, with significantly shorter RT in repetition trials (*M* = 788 ms, *SD* = 152 ms) as compared with switch trials (*M* = 956 ms, *SD* = 189 ms). All participants had longer mean RT in switch trials.

Th**e** interaction between *SNARC congruency* and *task switching* was not significant, *F*(1, 58) = 0.08, *p* = .78; η_p_² = .001. Hence, the SNARC effect was not significantly different between repetition trials (*M* = 17 ms, *SD* = 61 ms) and switch trials (*M* = 24 ms, *SD* = 87 ms). Congruent mean RTs were shorter in 35/59 participants (59.3%) in repetition trials and in 37/59 participants (62.7%) in switch trials. Bayesian analyses indicated a Bayes factor of BF_01_ = 0.849, suggesting that given the current data there is inconclusive evidence against a null effect of task switching on the SNARC effect.

### Modulation of the SNARC effect in magnitude comparison

Evaluating trials requiring magnitude comparisons only, a further ANOVA with the within-subject factors *SNARC congruency* (congruent vs incongruent) and *task switching* (repetition vs switch trials) was run on RTs. The main effect of *SNARC congruency* was significant, *F*(1, 58) = 7.76, *p* = .01; η_p_² = .12, indicating a regular SNARC effect with mean RTs being shorter for congruent trials (*M* = 821 ms, *SD* = 152 ms) as compared with incongruent trials (*M* = 858 ms, *SD* = 164 ms). The respective congruency effect was positive in 37/59 participants (62.7%). *Task switching* (repetition vs switch) also had a significant main effect on RTs, *F*(1, 58) = 164.75, *p* *<* .001; η_p_² = .74, with significantly shorter mean RT in repetition trials (*M* = 774 ms, *SD* = 140 ms) as compared with switch trials (*M* = 910 ms, *SD* = 176 ms). 57/59 (96.6%) of the participants had longer RT in switch trials.

The interaction between *SNARC congruency* and *task switching* was not significant, *F*(1, 58) = 0.94, *p* = .34; η_p_² = .02, indicating that the SNARC effect was not significantly different in repetition trials (*M* = 29 ms, *SD* = 92 ms) and switch trials (*M* = 53 ms, *SD* = 142 ms). Congruent RTs were shorter in 38/59 participants (64.4%) in repetition trials and in 40/59 participants (67.8%) in switch trials. Bayesian analyses revealed a Bayes factor of BF_01_ = 0.670. This indicates inconclusive evidence against a null effect of task switching on the SNARC effect.

## Discussion

In this study, we aimed to investigate whether previously observed effects of cognitive control on the numerical distance- and the SNARC effect generalise across different task combinations. Unlike previous task switching studies combining numerical with non-numerical tasks, we employed a purely numerical task switching paradigm requiring switches between magnitude comparisons and parity judgements. We observed the numerical distance effect to be reduced in switch trials. However, the SNARC effect was not modulated in switch trials. Considering the results of our sensitivity analysis, our study design only allowed us to detect a large interaction effect. Thus, one possible explanation for the observed lack of a modulation of the SNARC effect by task switching might be that this interaction effect—if present—was smaller than our study was able to detect. Hence, our study only partially supports the notion that cognitive control processes influence number processing.

As regards the numerical distance effect, it is important to note that the decreasing modulation observed in the current study contrasts with the increase of the distance effect in switch trials observed by Schliephake et al. (2020). This differential results pattern may indicate that the processing requirements of the specific tasks (i.e., numerical, input-output modality, letter judgement) might affect how cognitive control interacts with number processing. Similarly, a comparison of the results of our study (i.e., no evidence for an effect of cognitive control on the SNARC effect) with previous findings showing a modulation of the SNARC effect in settings requiring cognitive control (Pfister et al., 2013; Wendt et al., 2013) suggests that the influence of cognitive control on spatial numerical associations of numbers may also differ depending on task combinations. For instance, Wendt and colleagues (2013) found a significant modulation of the SNARC effect in a task switching paradigm involving letter classification and parity judgement tasks in a sample less than half of the present one (and thus lower statistical power).

Furthermore, Pfister and colleagues’ (2013) results also suggested that cognitive control may influence spatial numerical associations. They found that the congruency of the preceding stimulus modulated the SNARC effect. Interestingly, the methodological differences between these studies (task switching in Wendt et al., 2013, sequence congruity in Pfister et al., 2013) suggest that influences of cognitive control on number processing may depend on the actual task (combinations) at hand. Hence, in task switching situations, cognitive control seemingly has neither a uniform effect across different task combinations nor across numerical effects. Instead, both the combination of tasks used and the numerical effects under investigation seem to determine the interplay between numerical processing and cognitive control.

In the following, we suggest an information prioritisation account specifying when cognitive control processes modulate numerical effects. The foundation for this information prioritisation account is the degree of how explicitly the respective numerical information (e.g., magnitude/spatial associations) is processed in the employed tasks. In particular, one might speculate that cognitive control works as a prioritising mechanism that, depending on specific task requirements, allocates processing resources to the more explicitly processed information. For instance, in the current paradigm, number magnitude is processed explicitly in magnitude comparisons, while spatial associations of numbers (SNARC effect) as second -order association effects are not in the attentional focus. Hence, as processing numerical magnitude information is prioritised, it interacts with cognitive control processes. In contrast, spatial numerical associations of numbers represent a second-order association evolving from processing magnitude information (either explicitly in magnitude comparisons or implicitly in parity judgement tasks). As a consequence, the SNARC effect might be affected differently—in the present case, not significantly—by task switching-related cognitive control processes. In hindsight, at least two aspects must be considered when modulations of the SNARC effect in task switching paradigms are at stake. First, the SNARC effect has been reported to be more pronounced for longer reaction times (i.e., Cipora & Nuerk, 2013; see Cipora et al., 2019, for a review on potential measurement artefacts of the SNARC effect; Gevers et al., 2006). As such, previously observed larger SNARC effects in switch trials (e.g., Wendt et al., 2013) may be partially biased by longer reaction times. Another aspect to be considered is the hand-to-response assignment. In the current study, depending on the respective hand-to-response assignment, all magnitude comparisons are either SNARC compatible or SNARC incompatible. In particular, for the right large/even hand-to-response assignment, all responses in magnitude comparison trials are congruent with left-smaller/right-larger spatial numerical associations like the SNARC effect and vice versa. In turn, in trials requiring a switch from magnitude comparison to parity judgement, the SNARC congruency of hand-to-response assignment in the preceding magnitude comparison trial may have influenced the SNARC effect in the subsequent parity judgement trial. As such, these and potentially other influences suggest that the SNARC effect, as assessed in the current study, might not be the most well-suited numerical effect to evaluate influences of cognitive control on number processing.

Taking this into account, the idea for the information prioritisation account becomes clearer when comparing how explicit specific numerical information is processed for the tasks at hand. For instance, in both Schliephake and colleagues’ (2020) modality-compatibility task switch paradigm, as in our numerical task switch paradigm, numerical magnitude information had to be processed explicitly to perform the magnitude comparison task. Schliephake et al. (2020) found that the distance effect increased when input–output modality compatibility was switched (e.g., from compatible [i.e., verbal input − manual output] to incompatible [i.e., visual input − vocal output] or vice versa). This modulation fits nicely with our hypothesis that cognitive control acts as an information prioritising mechanism because in Schliephake et al. (2020) number magnitude information also had to be processed explicitly to perform the magnitude comparison task. Similarly, in the current study, magnitude information processing was seemingly prioritised because it had to be processed explicitly in magnitude comparisons. Hence, one would expect that number magnitude processing should be modulated in task switching paradigms (i.e., input–output modality compatibility switches; switching between number magnitude comparisons and parity judgements) as long as number magnitude needs to be processed explicitly.

While this account explains the observed modulation per se, interpreting the direction of the modulation of the distance effect (i.e., reduced in the current study vs increased in Schliephake et al., 2020) remains more speculative. The cause for the differential findings of Schliephake et al. (2020) and the current study may be that the current study required participants to switch between two numerical tasks—instead of switching between compatible and incompatible information processing channels when performing magnitude comparisons. Regarding Schliephake and colleagues’ (2020) findings, it seems reasonable that switching between different information processing channels (i.e., input–output modalities) might have interfered with magnitude processing efficiency. Hence, the observed larger distance effect may reflect less efficient processing of numerical magnitude (see the appendix in Moeller et al., 2011, for a discussion of how less efficient number magnitude processing can result in larger numerical distance effects). In turn, this might indicate that the information prioritisation mechanism engaged cognitive resources to compensate for the switch between input–output modalities that would otherwise be allocated to the processing of numerical magnitude. In the current study, however, the larger distance effect might indicate that processing number magnitude information was prioritised. In other words, rather than compensating for the interference of input–output modality switches (as in Schliephake et al., 2021), additional cognitive resources may have been allocated by cognitive control to (facilitate) number magnitude processing in switch trials. As a next step, additional studies are needed to evaluate further how the allocation of cognitive resources via cognitive control depends on task-specific requirements to understand better how cognitive control may act as an information prioritisation mechanism.

Regarding the SNARC effect, one might argue that, based on the suggested prioritisation account, spatial associations of numbers were processed more implicitly in our paradigm. Hence, spatial associations might not have been sufficiently prioritised to observe a modulation of the SNARC effect. Notably, the current study points towards an effect of specific task requirements on how cognitive control influences number processing, as previous studies, indicating an influence of cognitive control on the SNARC effect (i.e., Pfister et al., 2013; Wendt et al., 2013), employed different tasks or task combinations. For instance, Wendt and colleagues (2013) observed a smaller SNARC effect in a task switch paradigm when switching between letter classifications and parity judgements. The major difference of the study of Wendt and colleagues (2013) and the current study is that their study required domain switches between classifying letters and numbers (i.e., parity judgements). From an information prioritisation account perspective, one might argue that switches between domains, as required in the Wendt et al. (2013) study, may have caused stronger activation of attributes of the to be switched to domain, prioritising the respective information processing. In turn, this may have led to a more explicit processing of numerical information, as reflected by the significant modulation/reduction of the SNARC effect. The current study, however, only required switches within the number domain. Through these within-domain switches, one might assume a more balanced processing of specific numerical information (parity vs magnitude), which in turn prevented significant modulations of the SNARC effect compared with the study by Wendt et al. (2013) in which switches from the letter to the number domain may have led to specifically increased processing of respective number attributes.

Although past and present evidence can be synthesised in the suggested information prioritisation account, it still requires rigorous testing across different task combinations (numerical vs non-numerical but also numerical vs numerical) to evaluate systematically which task characteristics affect how number processing interacts with cognitive control. In particular, to further investigate how cognitive control acts as an information prioritisation mechanism, future research should explore the effect of task requirements (i.e., the automaticity of the tasks employed in the paradigm; switch or no task switch paradigm) on number processing.

Taken together, our study provides further evidence that number processing is influenced by cognitive control. However, it also indicates that further investigations are required to understand *how* cognitive control influences number processing. In particular, earlier and more general statements about cognitive control and number processing, such as “number processing is under cognitive control” (Macizo & Herrera, 2013) require refinement, as it seems that cognitive control rather flexibly adapts numerical information processing depending on a variety of factors, including task requirements such as which numerical information is processed explicitly.

## Supplemental Material

sj-docx-1-qjp-10.1177_17470218231154155 – Supplemental material for Influences of cognitive control on number processing: New evidence from switching between two numerical tasks Click here for additional data file. Supplemental material, sj-docx-1-qjp-10.1177_17470218231154155 for Influences of cognitive control on number processing: New evidence from switching between two numerical tasks by Andreas Schliephake, Julia Bahnmueller, Klaus Willmes, Iring Koch and Korbinian Moeller in Quarterly Journal of Experimental Psychology

## Notes

1. Error rates were within typical ranges in single-task (3.2% in magnitude comparisons, 4.2% in parity judgements) and task-switch blocks (6.2%). There were no indications of a speed accuracy trade-off.
