# Supplementary material for: Influences of cognitive control on number processing: New evidence from switching between two numerical tasks
Source: Q J Exp Psychol (Hove). 2023 Mar 7;76(11):2514–23. doi: 10.1177/17470218231154155 (PMC10585943; doi:10.1177/17470218231154155)
Supplement: sj-docx-1-qjp-10.1177_17470218231154155 – Supplemental material for Influences of cognitive control on number processing: New evidence from switching between two numerical tasks [file sj-docx-1-qjp-10.1177_17470218231154155.docx]

Supplementary Material for:

Influences of Cognitive Control on Number Processing: New Evidence from Switching Between two Numerical Tasks

Schliephake, A.^1^, Bahnmueller, J.^2^, Willmes, K.^3^, Koch, I.^4^, & Moeller, K^2,5,6^.

^1^ Leibniz-Institut für Wissensmedien, Tübingen, Germany

^2^ Centre for Mathematical Cognition, Loughborough University, Loughborough, UK

^3^ Department of Neurology, University Hospital, RWTH Aachen University, Aachen, Germany

^4^ Institute of Psychology, RWTH Aachen University, Aachen, Germany

^5^ Department of Psychology, University of Tübingen, Tübingen, Germany

^6^ LEAD Graduate School & Research Network, University of Tübingen, Tübingen, Germany

Corresponding author:

Andreas Schliephake

[a.schliephake@iwm-tuebingen.de](mailto:a.schliephake@iwm-tuebingen.de)

Leibniz-Institut für Wissensmedien, Tübingen, Germany

Schleichstraße 6, 72076

Tel.: +49 7071 979-264

In the following, please find a comparison of RT and ER means and standard deviations of the numerical distance effect and the SNARC effect in the single-task-, task repetition- and task switch condition (see Table 1, Table 2, and Table 3).

*Table 1*. Mean RTs (ms) and mean ERs (%) in magnitude comparisons with standard deviations depending on task type and numerical distance.

|  | **Single-Task** | **Task Repetition** | **Task Switch** |
| --- | --- | --- | --- |
| RT (ms) small distance | 546(100) | 801(145) | 929 (174) |
| RT (ms) large distance | 507(88) | 747 (141) | 891 (181) |
| ER (%) small distance | 5.7(2.3) | 6.3(4.9) | 4.4(5.2) |
| ER (%) large distance | 4.1(2.0) | 3.8(4.5) | 6.2(4.8) |
| **Distance Effects** (small - large) | | | |
| RT (ms) | 39 | 54 | 38 |
| ER (%) | 1.6 | 2.5 | -1.8 |

*Table 2*. Mean RTs (ms) and mean ERs (%) in parity judgements with the standard deviations (ms) depending on task type, and SNARC congruency.

|  | **Single-task** | **Task Repetition** | **Task Switch** |
| --- | --- | --- | --- |
| RT (ms) incongruent | 599(112) | 787(162) | 968(209) |
| RT (ms) congruent | 581(101) | 770(149) | 944(178) |
| ER (%) incongruent | 5.3(7.9) | 5.7(6.8) | 8.5(8.2) |
| ER (%) congruent | 3.0(3.4) | 6.0(9.3) | 8.4(9.9) |
| **SNARC Effects** (incongruent - congruent) | | | |
| RT (ms) | 18 | 17 | 24 |
| ER (%) | 2.3 | -0.3 | 0.1 |

*Table 3*. Mean RTs (ms) and mean ERs (%) in magnitude comparisons with the standard deviations (ms) depending on task type, and SNARC congruency.

|  | **Single-task** | **Task Repetition** | **Task Switch** |
| --- | --- | --- | --- |
| RT (ms) incongruent | 543(102) | 789(153) | 940(203) |
| RT (ms) congruent | 506(93) | 760(141) | 887(176) |
| ER (%) incongruent | 2.1(2.5) | 5.7(7.0) | 5.9(6.9) |
| ER (%) congruent | 2.7(6.6) | 4.4(3.7) | 4.7(5.2) |
| **SNARC Effects** (incongruent - congruent) | | | |
| RT (ms) | 37 | 29 | 53 |
| ER (%) | -0.6 | 1.3 | 1.2 |
